# Supplementary material for: Viability leads to the emergence of gait transitions in learning agile quadrupedal locomotion on challenging terrains
Source: Nat Commun. 2024 Apr 9;15:3073. doi: 10.1038/s41467-024-47443-w (PMC11271497; doi:10.1038/s41467-024-47443-w)
Supplement: Supplementary file 1 — Supplementary Information [file 41467_2024_47443_MOESM1_ESM.pdf]

Supplementary Information

Viability Leads to the Emergence of Gait Transitions in Learning Agile Quadrupedal Locomotion on Challenging Terrains

This file contains the following supplementary information:

Supplementary Method 1. Training details

Description: Reinforcement learning training details and parameters.

Supplementary Method 2. Reward Function Terms

Description: Reward function terms for flat terrain and gap crossing scenarios.

Supplementary Method 3. Viability and DCM Offset

Description: DCM and viability concept.

Supplementary Method 4. Robustness and Viability on Flat Terrain

Description: Phase coupling matrices for different gaits.

Supplementary Method 5. Role of RL in Flat Terrain Locomotion

Description: Supraspinal signals.

Supplementary Method 6. Phase coupling for different gaits

Description: Supraspinal signals.

Supplementary Method 7. Supraspinal Signals

Description: Supraspinal signals.

Supplementary Fig 1. Height-Map data around the robot in Isaac Gym.

Supplementary Fig 2. Inverted Pendulum, DCM, and viability kernel.

Supplementary Fig 3. DCM offset and maximum lateral external force.

Supplementary Fig 4. Output from the Reinforcement Learning-trained neural network.

Supplementary Fig 5. CPG amplitudes and phases of various limbs, modulated by the RL.

Supplementary Fig 6. Phase coupling for different gaits.

Supplementary Fig 7. Body pitch and roll orientation.

Supplementary Fig 8. CPG frequency, amplitude, and offset for the limbs.

Supplementary Table 1. Summary of experimental scenarios.

Supplementary Table 2. PPO Hyperparameters and neural network architecture used with PyBullet.

Supplementary Table 3. PPO Hyperparameters and neural network architecture used with Isaac Gym

Supplementary Table 4. Reward function term weights for flat terrain and gap-crossing scenarios.

Supplementary Method 1 Training details

We use PyBullet [1] and Isaac Gym [2] as our physics engines for training and simulation purposes, and the Unitree A1 and Go1 quadruped robots [3]. Supplementary Table 1 summarizes the training schemes for the flat terrain and gap crossing scenarios. To train the policies, we use Proximal Policy Optimization (PPO) [4], a state-of-the-art on-policy algorithm for solving the MDP, and Supplementary Table 2 shows the PPO hyperparameters and neural network architecture for PyBullet simulation. The control frequency of the policy is 100 Hz, and the torques computed from the desired joint positions are updated at 1 kHz. The equations for each of the oscillators (Eq. (1) and (2) in the main text) are thus also integrated at 1 kHz. The joint PD controller gains are $K_p = 100, K_d = 2$. All policies in PyBullet and Isaac Gym are trained for 3.5×10^7 and 36.4×10^7 samples respectively.

Supplementary Table 3 shows the PPO hyperparameters and neural network architecture used with Isaac Gym. Figure 1 illustrates the height-map as exteroceptive information that is used for training policies in Isaac Gym.

Supplementary Method 2 Reward Function Terms

Reward Function for Blind Locomotion on Flat Terrain: We design our reward function to promote viable states and fall prevention, tracking a desired base velocity, and minimizing energy expenditure as follows:

$$r_1 = \alpha_{1,flat} \cdot \min(f_x, d_{max}) + \alpha_{2,flat} \cdot exp\Big(-\frac{||\boldsymbol{v}_{des} - \boldsymbol{v}_{real}||^2}{0.25}\Big) + \alpha_{3,flat} \cdot |\boldsymbol{\tau} \cdot (\dot{\boldsymbol{q}}^t - \dot{\boldsymbol{q}}^{t-1})| + \alpha_{4,flat} \cdot ||\boldsymbol{o}_{base} - \boldsymbol{o}_{zero}||)$$

- *Forward progress (promoting viability)*: In the first term, f_x corresponds to forward progress in the world (along the x-direction). This term promotes viability of the system, as continuous high forward progress indicates that the robot has not fallen. Additionally in r_2 below, forward progress in a gap crossing scenario indicates that the robot is able to traverse the gaps without falling. We limit this term to avoid exploiting simulator dynamics and achieving unrealistic speeds, where d_{max} is the maximum distance/reward the robot can receive for moving forward over the last control cycle ($\alpha_{1,flat} = 0.03$).

- *Velocity tracking reward*: In the second term, $\boldsymbol{v}_{des}$ and $\boldsymbol{v}_{real}$ correspond to desired and real (actual) locomotion velocity in the world x-direction. This term encourages the robot to follow the desired locomotion velocity. This term also promotes viability of the system, as tracking the steady state velocity indicates that the robot has not fallen ($\alpha_{2,flat} = 0.03$).

- *Power*: The fourth term penalizes power in order to find energy efficient gaits, where $\dot{\boldsymbol{q}}$ and $\boldsymbol{\tau}$ are vectors of all of the joint velocities and torques ($\alpha_{3,flat} = -0.00001$).

- *Base orientation penalty*: The third term penalizes body orientation deviations from the nominal base horizontal configuration ($\alpha_{4,flat} = -0.02$). Large roll and pitch angles increase the likelihood of falling,

thus penalizing these occurrences helps to prevent the system from getting closer to the boundary of the viability kernel.

Reward Function for Gap Crossing: To analyze the effects of the different reward function terms, for gap crossing scenarios we consider the following reward function r_2 with varying zero, low, medium, and high weights, summarized in Supplementary Table 4. The low and medium weights are selected to be approximately 10% and 50% of the high values, respectively.

$$r_2 = \alpha_{1,gap} \cdot \min(f_x, d_{max}) + \alpha_{2,gap} \cdot \sum_{i=1}^{4} \max(0, F_{c,i} - F_{c,max}) + \alpha_{3,gap} \cdot |\boldsymbol{\tau} \cdot (\dot{\boldsymbol{q}}^t - \dot{\boldsymbol{q}}^{t-1})| + \alpha_{4,gap} \cdot ||\boldsymbol{o}_{base} - \boldsymbol{o}_{zero}||$$

- *Forward progress (promoting viable states)*: Regarding forward progress, we assign 8.0, 4.0, 0.8, and 0 as the high, medium, low, and zero values, respectively, for the parameter $\alpha_{1,gap}$.
- *Peak contact force*: To penalize the peak contact reaction forces, we assign −0.01, −0.005, −0.001, 0 as high, medium, low, and zero weights, respectively, for the parameter $\alpha_{2,gap}$. The determination of the maximum contact force, denoted as $F_{c,max} = 180N$, was accomplished through monitoring the contact reaction force during locomotion on flat terrain at a speed of 1.2 m/s.
- *Power*: For penalizing power, we assign −0.001, −0.0005, −0.0001, and 0 as the high, medium, low, and zero weights, respectively, for the parameter $\alpha_{3,gap}$.
- *Base orientation penalty*: For penalizing the body orientation deviations, we use $\alpha_{4,gap} = -0.25$.

Supplementary Method 3 Viability and DCM Offset

The viability kernel encompasses all states from which the mobile system can avoid falling or colliding with obstacles through corrective control actions. Outside of this set, falling is unavoidable (Supplementary Fig. 2).

In the following sections, we will conduct the viability analysis based on the inverted pendulum model and the Divergent Component of Motion (DCM) concept.

Supplementary Method 3.1 Linear Inverted Pendulum

The LIPM has been widely utilized to describe the dynamics of the CoM for bipedal locomotion [5]. The LIPM assumes a constant rate of change of centroidal angular momentum and movement of the CoM height within a plane. Based on the assumptions, the equations of motion for the LIPM can be derived as follows:

$$\ddot{\mathbf{x}} = \omega^2(\mathbf{x} - \mathbf{cop}) \qquad \textbf{(Supplementary Equation. 1)}$$

in which $\mathbf{x} = [x_{com}, y_{com}]^T$ is the horizontal position of the CoM, $\omega_0 = \sqrt{\frac{g}{\Delta z}}$ is the natural frequency of the LIPM, and $\mathbf{cop} = [cop_x, cop_y]^T$ is the horizontal position of the center of pressure (CoP).

Supplementary Method 3.2 Divergent Component of Motion (DCM)

In this section, we provide an overview of the DCM concept's background. The dynamics of the CoM, as modeled by the LIPM, can be split into stable and unstable components [6, 7, 8]. The unstable component is referred to as the DCM and is defined as follows:

$$\boldsymbol{\xi} = \mathbf{x} + \frac{\dot{\mathbf{x}}}{\omega} \qquad \text{(\textbf{Supplementary Equation. 2})}$$

From (**Supplementary Equation. 2**), the CoM dynamics is given by:

$$\dot{\mathbf{x}} = \omega(\boldsymbol{\xi} - \mathbf{x}) \qquad \text{(\textbf{Supplementary Equation. 3})}$$

By differentiating (**Supplementary Equation. 2**) and substituting (**Supplementary Equation. 1**), the DCM dynamics is expressed as:

$$\dot{\boldsymbol{\xi}} = \omega(\boldsymbol{\xi} - \mathbf{cop}) \qquad \text{(\textbf{Supplementary Equation. 4})}$$

Supplementary Fig. 2 illustrates the relationship between DCM dynamics, CoM, and the CoP. By re-arranging DCM dynamics (**Supplementary Equation. 4**), the following ordinary differential equation (ODE) holds:

$$\dot{\boldsymbol{\xi}} - \omega\boldsymbol{\xi} = -\omega\, \mathbf{cop}_0 \qquad \text{(\textbf{Supplementary Equation. 5})}$$

The solution to (**Supplementary Equation. 5**) can be written as:

$$\boldsymbol{\xi}(t) = e^{\int \omega dt}\left[\int (-\mathbf{cop}_0\, \omega)\, e^{\int -\omega dt} dt + \mathbf{C}\right], \qquad \text{(\textbf{Supplementary Equation. 6})}$$

where $C \in \mathbb{R}^2$ is the vector of unknown coefficients that can be found by imposing the boundary conditions. Therefore, we can find these coefficients by solving the problem (**Supplementary Equation. 6**) either as an initial value problem, namely

$$\boldsymbol{\xi}(0) = \boldsymbol{\xi}_0 = \mathbf{cop}_0 + \mathbf{C}_0, \qquad \text{(\textbf{Supplementary Equation. 7})}$$

or as a final value problem:

$$\boldsymbol{\xi}(T) = \boldsymbol{\xi}_T = \mathbf{cop_0} + \mathbf{C_f}\, e^{\omega T}. \qquad \text{(\textbf{Supplementary Equation. 8})}$$

Therefore, by solving the equation (**Supplementary Equation. 4**) as an initial value problem, we arrive at the following equation that represents the time evolution of the DCM:

$$\boldsymbol{\xi} = (\boldsymbol{\xi}_0 - \mathbf{cop_0})\exp(\omega t) + \mathbf{cop_0} \qquad \text{(\textbf{Supplementary Equation. 9})}$$

We also can solve the CoM dynamics (**Supplementary Equation. 3**) by treating it as an initial value problem:

$$\mathbf{x} = (\mathbf{x_0} - \boldsymbol{\xi}_0)\exp(-\omega t) + \boldsymbol{\xi}_0 \qquad \text{(\textbf{Supplementary Equation. 10})}$$

As evident from the above equation, the CoM exhibits stable dynamics, with the exponential term being negative. However, the DCM exhibits unstable dynamics, characterized by a positive exponential term. This indicates that the difference between ξ_0 and cop_0 increases exponentially over time. The distance between the CoP and the DCM is referred to as the DCM offset, and minimizing this distance is crucial for maintaining viable states.

To find a DCM trajectory that satisfies both the initial and the final condition problems, the coefficient C_0 must equal C_f. Thus, by combining (**Supplementary Equation. 7**) and (**Supplementary Equation. 8**), we have:

$$\boldsymbol{\xi}_0 - \mathbf{cop}_0 = (\boldsymbol{\xi}_T - \mathbf{cop}_0)\, e^{-\omega T}. \qquad \text{(Supplementary Equation. 11)}$$

Now by defining $\sigma = e^{\omega T}$ we obtain :

$$\boldsymbol{\xi}_T + \mathbf{cop}_0(-1+\sigma) - \boldsymbol{\xi}_0\sigma = 0. \qquad \text{(Supplementary Equation. 12)}$$

Let $\mathbf{cop}_T$ represent the CoP position at the start of the next step, and $\boldsymbol{\gamma_T} = \boldsymbol{\xi_T} - \mathbf{cop_T}$ denote the DCM offset for the next step (i.e, the end of this step) and $\boldsymbol{\gamma_0} = \boldsymbol{\xi_0} - \mathbf{cop_0}$ denote the current DCM offset. Therefore, straightforward calculations lead to:

$$\boldsymbol{\gamma_T} + \mathbf{cop}_T - \boldsymbol{\gamma_0}.\sigma = \mathbf{cop}_0. \qquad \text{(Supplementary Equation. 13)}$$

We will use this equation to determine the viability kernel for the LIPM.

Supplementary Method 3.3 Viability bound on the DCM offset

We now express the viability region of the LIPM [9, 10, 11, 12] in terms of the DCM offset. Computing the viability kernel is generally intractable, but fortunately it is possible to characterize these bounds for the LIPM as it was shown in [10, 9]. It is noteworthy this viability analysis relies on the LIPM assumptions which assume a constant centroidal angular momentum, movement of the CoM height within a plane, and enough available coefficient of friction, which may not hold during high speed locomotion where the legs generate high angular momentum and the CoM height can have higher acceleration.

In the (**Supplementary Equation. 13**), γ_T is the DCM offset for the next steps, $\mathbf{cop}_T$ is the CoP position for the next step and $L_{step} = \mathbf{cop}_T - \mathbf{cop}_0$ indicates the step length. Therefore, the maximum possible available value for L_{step} is the L_{max}, which is the maximum feasible step length of the robot. $\mathbf{cop}_0$ indicates the current step position and current CoP. $\sigma = e^{\omega T}$ indicates the step duration that fastest step possible has $\sigma_{min} = e^{\omega T_{min}}$ that is found based on actuation power of the robot.

By writing the (**Supplementary Equation. 13**) based on step length, we have:

$$L_{step} = -\boldsymbol{\gamma_T} + \boldsymbol{\gamma_0}.\sigma \qquad \text{(Supplementary Equation. 14)}$$

We now describe the viability boundary using the DCM offset. We limit our analysis to the sagittal plane

dynamics for forward walking as the analysis for backward walking is similar. The maximum DCM offset γ_{max} is linked to the maximum step length and minimum step duration by the following relationship:

$$\boldsymbol{\gamma_{max}} = \frac{L_{max}}{\sigma_{min} - 1} \qquad \textbf{(Supplementary Equation. 15)}$$

This maximum offset serves as a crucial threshold, distinguishing between viable and non-viable states: I) if the DCM offset is larger than γ_{max}, every potential combination of step timing and location will result in divergence and fall and II) if the DCM offset is smaller than (or equal to) γ_{max}, there is at least one possible combination of step timing and position that prevents the DCM from diverging (falling).

If $\boldsymbol{\gamma_0} > \boldsymbol{\gamma_{max}}$ at the beginning of a step then we have:

$$\boldsymbol{\gamma_0} = \boldsymbol{\gamma_{max}} + \epsilon \qquad \textbf{(Supplementary Equation. 16)}$$

where $\epsilon > 0$. Using (**Supplementary Equation. 14**) and (**Supplementary Equation. 16**), for the DCM offset at the end of the step, we have:

$$\boldsymbol{\gamma_T} = -L_{step} + \boldsymbol{\gamma_{max}}.\sigma + \epsilon.\sigma \qquad \textbf{(Supplementary Equation. 17)}$$

By substituting $L_{step} = L_{max}$ and $\sigma = \sigma_{min}$, we can determine the minimum realizable DCM offset $\gamma_{x,T}$:

$$\boldsymbol{\gamma_T} = \boldsymbol{\gamma_{max}} + \epsilon.\sigma_{min} \qquad \textbf{(Supplementary Equation. 18)}$$

Therefore, we observe that the minimum realizable DCM offset at the end of the step grows by $\epsilon.\sigma_{min}$. Consequently, a series of steps will result in a diverging geometric series with a ratio of σ_{min}, implying that all possible choices of step location and timing will lead to divergence, ultimately resulting in a fall.

Supplementary Method 4 Robustness and Viability on Flat Terrain

In the preceding section, we noted that augmenting the DCM offset moves the system closer to the boundary of the viability kernel. Moreover, there exists a maximum DCM offset beyond which the system state becomes non-viable. Conversely, reducing the DCM offset moves the states of the system further inside the viability kernel, indicating an improvement in viability. Nevertheless, the DCM offset analysis in the preceding section relied on assumptions inherent to the linear inverted pendulum model (LIPM). The LIPM assumes zero change in angular momentum around the CoM, a constant CoM height, and enough available coefficient of friction. Although this model is suitable for low-speed locomotion, its applicability becomes more questionable for high-speed locomotion scenarios.

Viability on flat terrain for a quadruped robot can be quantified by the maximum external push the robot can tolerate without falling. For example, consider two walking gaits, both deemed viable. However, their robustness under external pushes may differ, with one being able to tolerate higher forces. This observation

suggests that the latter gait exhibits better viability conditions. Our analysis of gait changes from walking to trotting on flat terrain revealed a reduction in lateral DCM offset. We hypothesize that the maximum external push a robot can withstand without falling will decrease after changing the gait at a specific speed. It is important to note that the DCM offset in the longitudinal direction will increase with speed for all gaits, as the CoM and DCM need to undergo faster and more dynamic forward locomotion. However, for forward locomotion, we desire a lateral DCM offset of zero since there is no intended lateral speed. In the following section, we will further explore the relationship between the DCM offset and external pushes.

We conducted simulations consisting of 350,000 samples, equivalent to an average of 50 tests of 7 seconds of locomotion each, applying external lateral pushes at various locomotion speeds for two policies trained for walking and trot gaits. In these simulations, a lateral external push was initiated at a random moment and lasts for 0.7 second. The falling state is identified when the robot reaches a height of less than 15 cm. The simulation is executed at various speeds, and the outcomes regarding the maximum applied force are illustrated in Supplementary Figure (3). As evidenced by the transition from a walk to a trot gait at the energetically optimal speed, there is a noticeable increase in the maximum external force that the robot can tolerate without falling. This observation aligns with the changes in DCM offset, with a reduction in DCM offset after transitioning from a walk to a trot gait. This reduction signifies an improvement in viability after the gait transition. In the walk gait, as the speed increases, a consistent correlation is observed between DCM offset and the maximum applied force. Conversely, in the trot gait, there is an overall reduction in the maximum force applied, aligning with the increasing DCM offset within the velocity range of 0.8 $\mathrm{m\,s^{-1}}$ to 1.2 $\mathrm{m\,s^{-1}}$. However, beyond a velocity of 1.2 $\mathrm{m\,s^{-1}}$, the maximum external forces show an increase that is not consistently in line with the escalating DCM offset during this speed interval. This discrepancy may be attributed to the assumptions inherent in the LIPM and DCM viability analysis, which rely on a zero centroidal angular momentum, constant body height, and available enough friction. These assumptions do not hold for high-speed velocities where limb rotation occurs rapidly.

Supplementary Method 5 Role of RL in Flat Terrain Locomotion

In this work, we employ Reinforcement Learning (RL) to train a neural network for modulating the frequency and amplitude of Central Pattern Generators (CPG). The primary objective of the Deep-RL approach is to optimize the actions of the deep neural network to maximize a reward function. In our specific context, the reward function on flat terrain aims to enhance viability while penalizing energy inefficiency and deviation from the desired velocity. In particular, the ability to generate viable locomotion behavior for different velocities using a single neural network underscores the pivotal role of Reinforcement Learning (RL). Furthermore, in scenarios involving high-speed locomotion, the continuous adaptation of CPG frequency and amplitude proves beneficial in coordinating phase changes during both stance and swing phases.

To underscore the role of Reinforcement Learning (RL) in flat terrain locomotion within the proposed CPG-RL framework, simulations are conducted by fixing the output of the RL-trained neural network as the input

to the CPG.

We use the trotting gait policy as shown in Figure (2)-a of the manuscript, setting the locomotion speed to 2.2 $\mathrm{m\,s^{-1}}$. The neural network generates time-varying (based on sensory feedback) output representing the desired amplitude and frequency of the CPG. Subsequently, three random instances of this output are selected, and we fix the parameters of the CPG with these values so that they are no longer modulated. The simulation results indicate a failure to move forward in case 1, and falls in cases 2 and 3, which highlight the contribution of the RL-trained neural network.

In Supplementary Figure 4-a, the output of the neural network for the trotting policy is depicted, showcasing the desired amplitude and frequency of the CPG. The output of the neural network is normalized between -1 and 1, which is a common practice in deep reinforcement learning. Supplementary Figure 4-b presents snapshots of normal locomotion driven by the neural network and three distinct cases with fixed CPG inputs, illustrating locomotion failures with fixed CPG inputs.

Furthermore, Supplementary Figure 5-a illustrates the constantly changing CPG phase and real amplitude, adapting based on the stance/swing phase of the limbs, where the shadow bar denotes the stance phase. Conversely, fixing the CPG frequency and amplitude results in a lack of adaptive behavior, as evidenced by the absence of such fluctuations.

Supplementary Method 6 Phase coupling for different gaits

The coupling matrix $\mathbf{\Phi}$ representing a walk, trot and bound gaits can be defined as:

$$\mathbf{\Phi}_{gait} = \begin{bmatrix} 0 & -A & -B & -C \\ A & 0 & A-B & A-C \\ B & B-A & 0 & B-C \\ C & C-A & C-B & 0 \end{bmatrix} \qquad \text{(\textbf{Supplementary Equation. 19})}$$

where the row/column order is Front Left (FL), Front Right (FR), Hind Left (HL), Hind Right (HR) and A, B and C are defined in Supplementary Fig. 6.

Supplementary Method 7 Supraspinal Signals

Figure 8 shows the supraspinal drive performing complex interlimb coordination by modulating each limb's frequency, amplitude, and foot position offset in order to successfully cross eight consecutive gaps. On average, the limb frequency increases to near the maximum for all legs before/after crossing each of the gaps (shadow bars). The amplitude for the front limbs distinctly increases while over the gap in order to take a larger step, which together with the offset terms are also important components for interlimb coordination. The offset terms go from positive hip x offsets to negative hip x offsets to help cross the gaps for all limbs.

Supplementary Table 1: Summary of experimental scenarios.

Options \ Exp Name	**Flat Terrain**	**Gap Crossing**
Simulator	PyBullet	PyBullet/Isaac Gym
Animal Data	✓	×
Investigated Criteria	Viability/Energy	Viability/Contact Peak Force/Energy
Neural gait coupling	Walk/Trot	Without Coupling
Blind Observation space	✓	✓
$\mathbf{a}_{off} = [\mathbf{x}_{off}] \in \mathbb{R}^4$ in Action space	×	✓
$\mathbf{a}_{osc} = [\mu, \omega] \in \mathbb{R}^8$ in Action space	✓	✓
Exteroceptive Observation Space	×	LiDAR/Explicit Features/Height Map
Reward for Minimizing Energy	✓	✓
Reward for Viability	✓	✓
Reward for Peak Contact Force	×	✓
Reward for Velocity Tracking	✓	×
Reward for Penalizing Body Orientation	✓	✓

Supplementary Table 2: PPO Hyperparameters and neural network architecture used with PyBullet.

Parameter	Value	Parameter	Value
Batch size	4096	GAE discount factor	0.95
Mini-bach size	128	Desired KL-divergence kl^*	0.01
Number of epochs	10	Learning rate α	1e-4
Clip range	0.2	NN Hidden Layers	[256,256]
Entropy coefficient	0.01	Activation	tanh
Discount Factor	0.99	Framework	Torch

Supplementary Table 3: PPO Hyperparameters and neural network architecture used with Isaac Gym.

Parameter	Value	Parameter	Value
Batch size	98304 (4096x24)	GAE discount factor	0.95
Mini-bach size	24576 (4096x6)	Desired KL-divergence kl^*	0.01
Number of epochs	5	Learning rate α	adaptive
Clip range	0.2	NN Hidden Layers	[512, 256, 128]
Entropy coefficient	0.01	Activation	elu
Discount factor	0.99	Framework	Torch

Supplementary Table 4: Reward function term weights for flat terrain and gap-crossing scenarios.

Reward function term weights				
Terms	Flat Terrain	Gap Terrain		
Viability	$\alpha_{1,flat} = 0.03$	$\alpha_{1,gap,low} = 0.8$	$\alpha_{1,gap,medium} = 4.0$	$\alpha_{1,gap,high} = 8.0$
Velocity Tracking	$\alpha_{2,flat} = 0.03$	$\times$		
Peak Contact Force	$\times$	$\alpha_{2,gap,low} = -0.001$	$\alpha_{2,gap,medium} = -0.005$	$\alpha_{2,gap,high} = -0.01$
Power	$\alpha_{3,flat} = -0.00001$	$\alpha_{3,gap,low} = -0.0001$	$\alpha_{3,gap,medium} = -0.0005$	$\alpha_{3,gap,high} = -0.001$
Base Orientation	$\alpha_{4,flat} = -0.02$	$\alpha_{4,gap} = -0.25$		

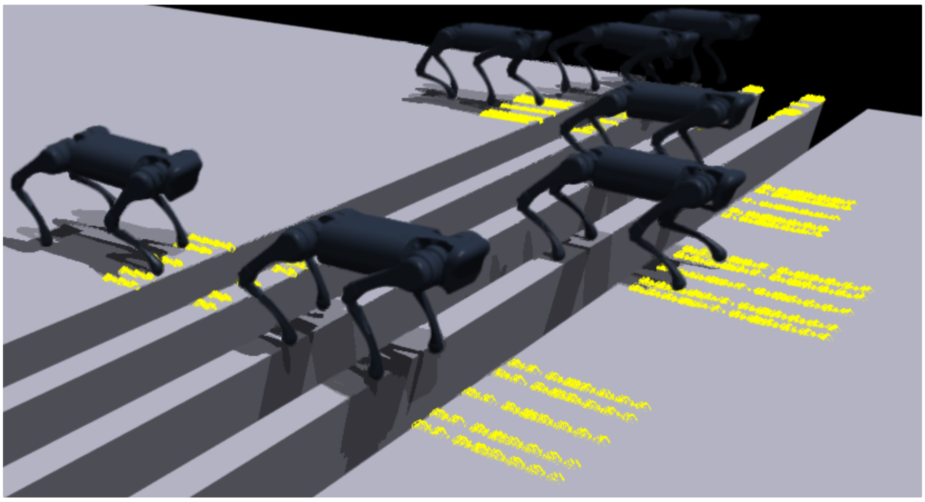

Supplementary Fig 1: Height-Map data around the robot in Isaac Gym.

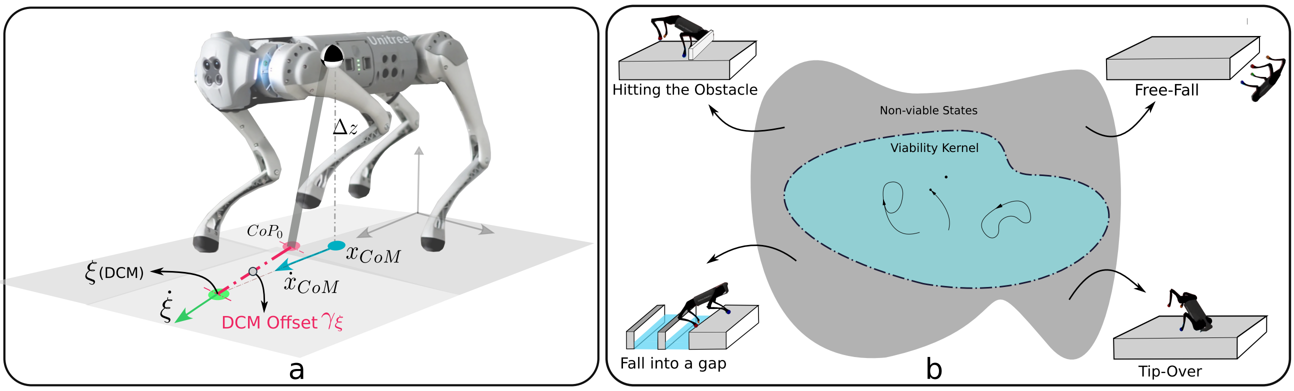

Supplementary Fig 2: **a:** Inverted Pendulum and DCM, **b:** Viability Kernel [12]

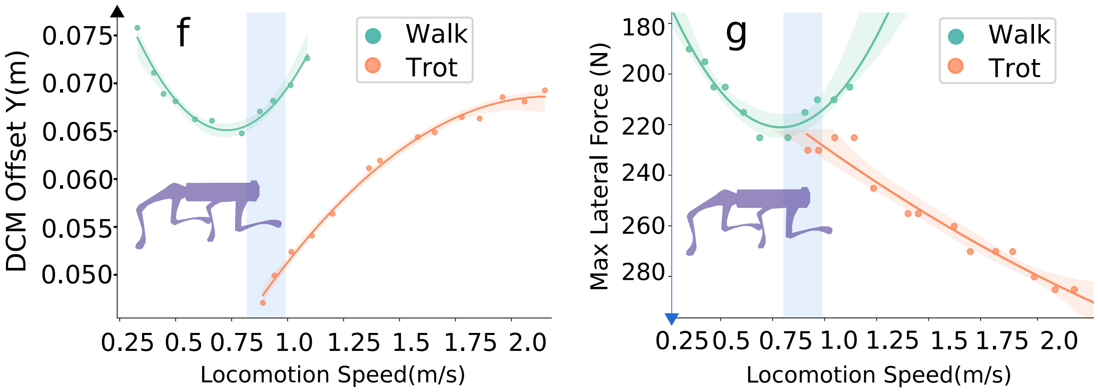

Supplementary Fig 3: DCM offset and maximum lateral external force. In panels (f) and (g) of Figure 2 in the main text, the (f) shows the Lateral DCM Offset, while (g) illustrates the maximum lateral push that the robot can withstand before falling. Please note that the vertical axis of (g) is reversed for easier comparison with (f).

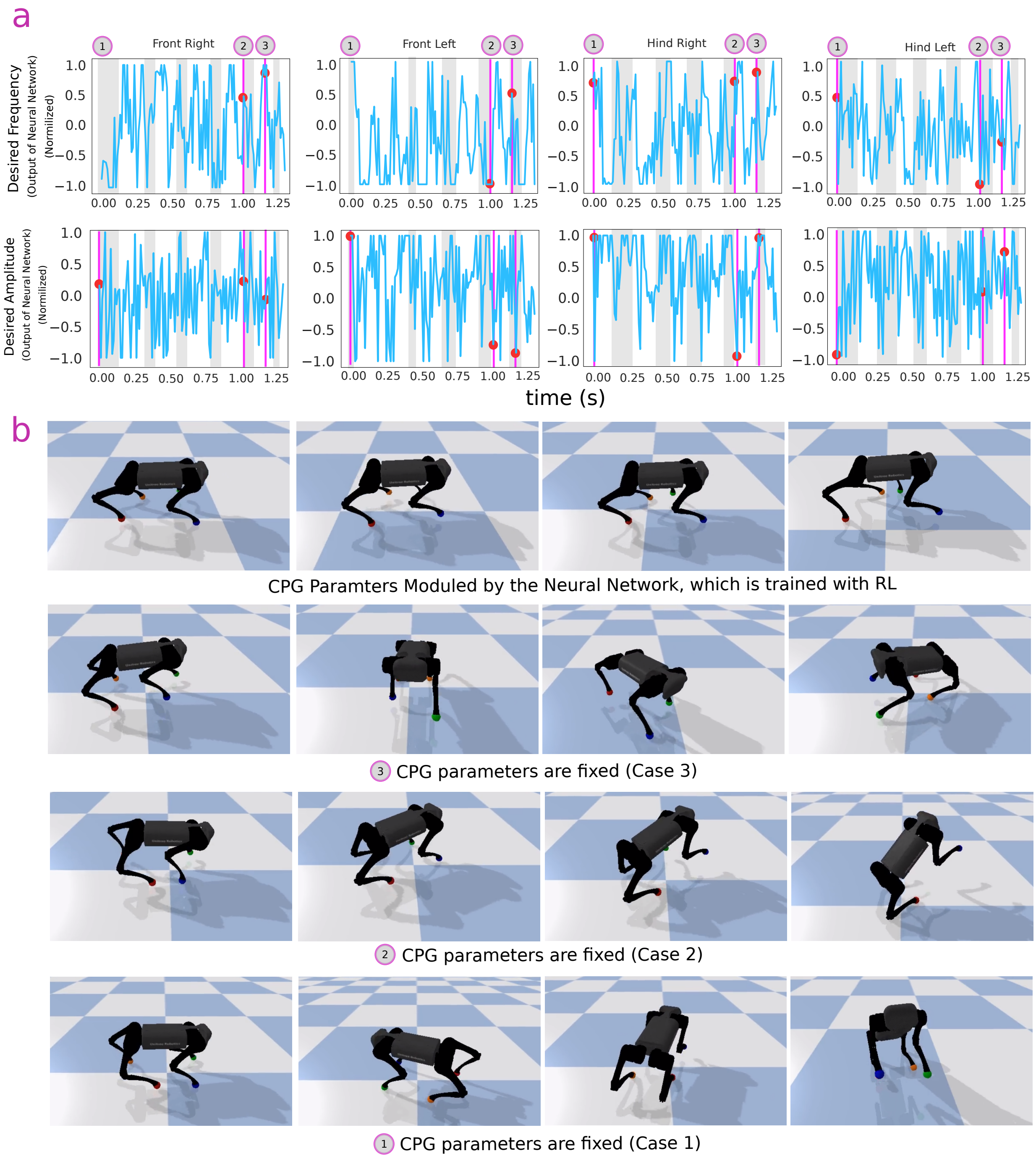

Supplementary Fig 4: **a.** Output from the Reinforcement Learning-trained neural network, which corresponds to the neural network policy depicted in Figure (2)-a. The locomotion speed is set at $m.s^{-1}$. **b.** Cases 1, 2, and 3 are randomly selected, each representing a fixed desired CPG amplitude and frequency. This selection aims to explore the impact of the RL-trained neural network on locomotion. As illustrated in the snapshots of these cases, it is evident that the robot fails to achieve forward motion that highlights the importance of the RL on the flat terrain.

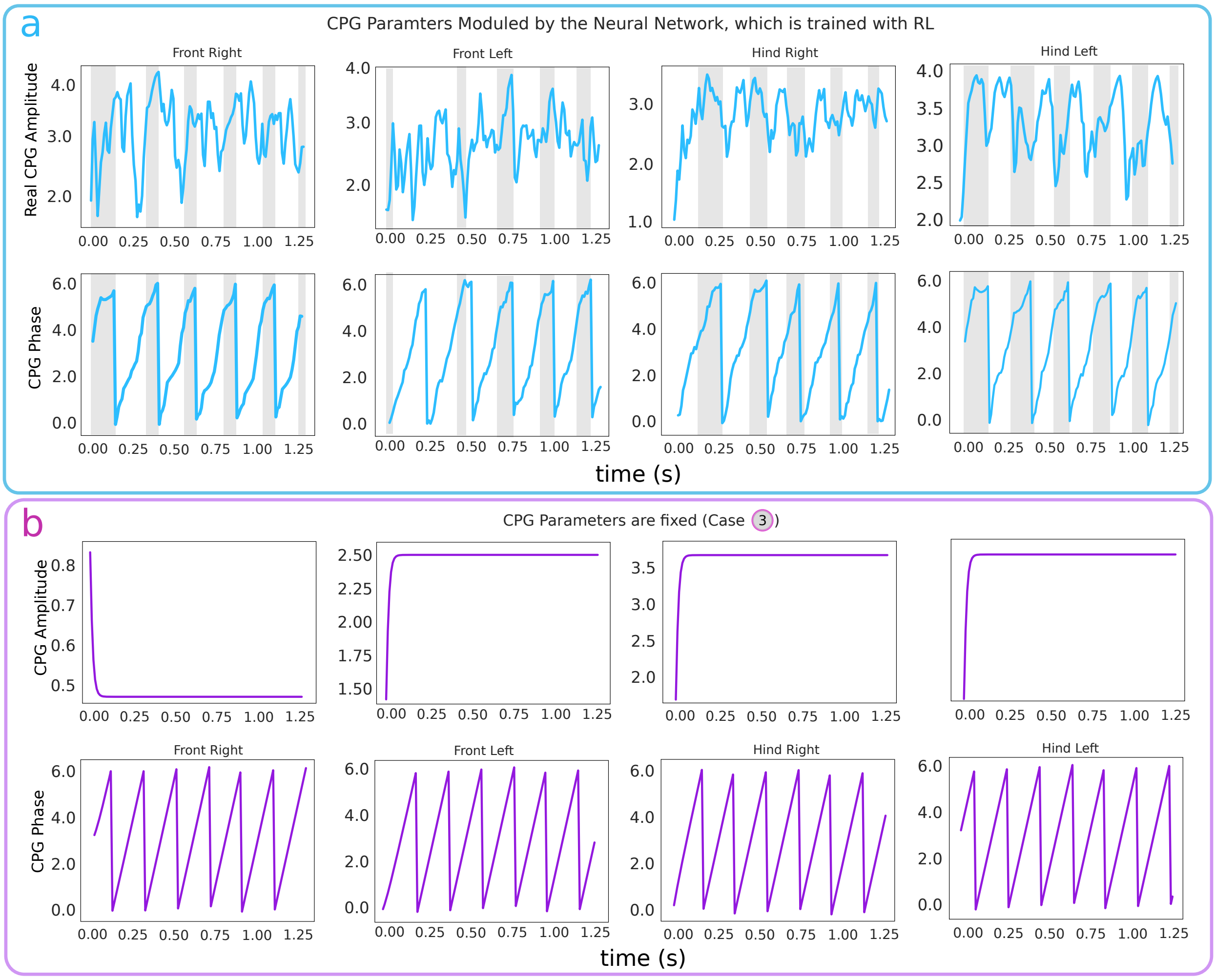

Supplementary Fig 5: **a.** The real CPG amplitudes and phases of various limbs, modulated by the Reinforcement Learning-trained neural network, contribute to the viability of locomotion at a speed of 2.2 m.s^{-1} (These plots correspond to the Supplementary Figure 4-a). The shadow bar visually represents the stance phase of each limb, demonstrating the adaptive changes in phase based on the stance/swing mode of the limbs. **b.** CPG amplitude and phases of the different limbs of case 3 in Supplementary Figure 4. In this scenario, where the CPG parameters are fixed, the absence of adaptive behavior in the phase changes during the swing/stance phases results in nonviable locomotion, as demonstrated in Supplementary Figure.4-b.

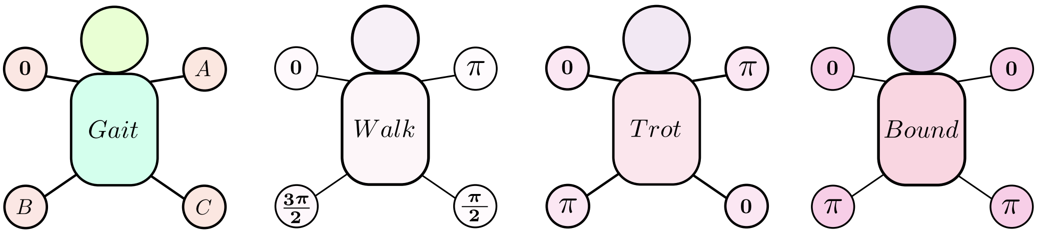

Supplementary Fig 6: Phase coupling for different gaits.

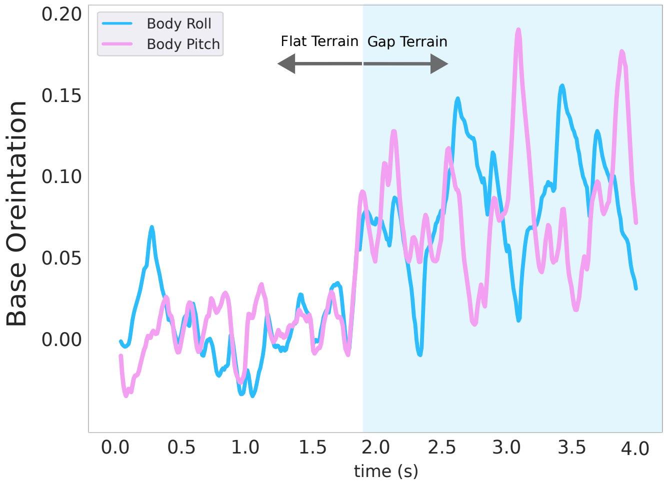

Supplementary Fig 7: Body pitch and roll orientation are pertinent to the gap-crossing experiments depicted in Figure 3. The robot's roll and pitch angles undergo abrupt changes upon reaching the gaps. In this scenario, the roll angle increases during gap crossing since there are no symmetry constraints imposed on the body. These roll angles elucidate why the Hind Left (HL) limb in Figure 3-b exhibits a greater height.

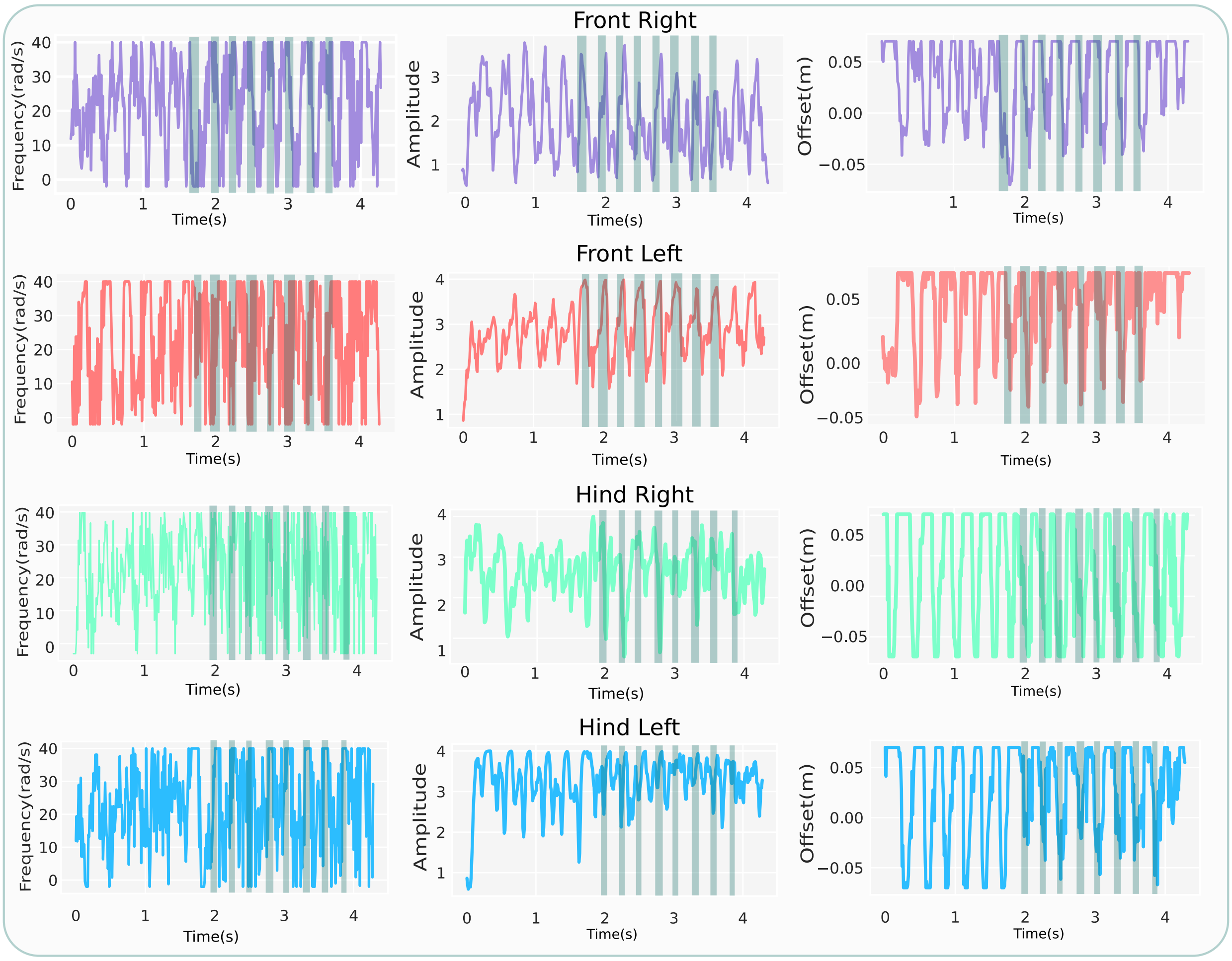

Supplementary Fig 8: CPG frequency, amplitude, and offset for the limbs. The shadow bars indicate when the foot is over a gap. The supraspinal drive performing complex interlimb coordination by modulating each limb's frequency, amplitude, and foot position offset in order to successfully cross eight consecutive gaps. On average, the limb frequency increases to near the maximum for all legs before/after crossing each of the gaps (shadow bars). The amplitude for the front limbs distinctly increases while over the gap in order to take a larger step, which together with the offset terms are also important components for interlimb coordination. The offset terms go from positive hip x offsets to negative hip x offsets to help cross the gaps for all limbs.

References

[1] Erwin Coumans and Yunfei Bai. Pybullet, a python module for physics simulation for games, robotics and machine learning. `http://pybullet.org`, 2016–2019.

[2] Viktor Makoviychuk, Lukasz Wawrzyniak, Yunrong Guo, Michelle Lu, Kier Storey, Miles Macklin, David Hoeller, Nikita Rudin, Arthur Allshire, Ankur Handa, et al. Isaac gym: High performance gpu-based physics simulation for robot learning. *arXiv preprint arXiv:2108.10470*, 2021.

[3] Unitree Robotics. A1, February 2021.

[4] John Schulman, Philipp Moritz, Sergey Levine, Michael I. Jordan, and Pieter Abbeel. High-dimensional continuous control using generalized advantage estimation. *CoRR*, abs/1506.02438, 2015.

[5] Shuuji Kajita, Fumio Kanehiro, Kenji Kaneko, Kiyoshi Fujiwara, Kensuke Harada, Kazuhito Yokoi, and Hirohisa Hirukawa. Biped walking pattern generation by using preview control of zero-moment point. In *International Conference on Robotics and Automation*, volume 2, pages 1620–1626. IEEE, 2003.

[6] Milad Shafiee-Ashtiani, Aghil Yousefi-Koma, Masoud Shariat-Panahi, and Majid Khadiv. Push recovery of a humanoid robot based on model predictive control and capture point. In *2016 4th International Conference on Robotics and Mechatronics (ICROM)*, pages 433–438. IEEE, 2016.

[7] Milad Shafiee-Ashtiani, Aghil Yousefi-Koma, and Masoud Shariat-Panahi. Robust bipedal locomotion control based on model predictive control and divergent component of motion. In *2017 IEEE International Conference on Robotics and Automation (ICRA)*, pages 3505–3510. IEEE, 2017.

[8] Zeynep Özge Orhan, Milad Shafiee, Vincent Juillard, Joel Coelho Oliveira, Auke Ijspeert, and Mohamed Bouri. Exorecovery: Push recovery with a lower-limb exoskeleton based on stepping strategy. *arXiv e-prints*, pages arXiv–2310, 2023.

[9] Mohammad Hasan Yeganegi, Majid Khadiv, Andrea Del Prete, S Ali A Moosavian, and Ludovic Righetti. Robust walking based on mpc with viability guarantees. *IEEE Transactions on Robotics*, 38(4):2389–2404, 2021.

[10] Majid Khadiv, Alexander Herzog, S Ali A Moosavian, and Ludovic Righetti. Walking control based on step timing adaptation. *IEEE Transactions on Robotics*, 36(3):629–643, 2020.

[11] Pierre-Brice Wieber. On the stability of walking systems. In *Proceedings of the international workshop on humanoid and human friendly robotics*, 2002.

[12] Pierre-Brice Wieber. Viability and predictive control for safe locomotion. In *2008 IEEE/RSJ International Conference on Intelligent Robots and Systems*, pages 1103–1108. IEEE, 2008.
