# Supplementary material for: Viability leads to the emergence of gait transitions in learning agile quadrupedal locomotion on challenging terrains
Source: Nat Commun. 2024 Apr 9;15:3073. doi: 10.1038/s41467-024-47443-w (PMC11271497; doi:10.1038/s41467-024-47443-w)
Supplement: Supplementary file 3 — Description of additional supplementary files [file 41467_2024_47443_MOESM3_ESM.pdf]

DESCRIPTION OF ADDITIONAL SUPPLEMENTARY FILES DOCUMENT

Supplementary Movie 1. Hardware experiments of Gait transition (Figure 7).

Supplementary Movie 2. Gap Crossing in Pybullet (Figure 3).

Supplementary Movie 3. Gap Crossing in Isaac-Gym, Reward function analysis (Figure 4).

Supplementary Movie 4. Gap Crossing in Pybullet, Observation space and gait coupling analysis, (Figure 5).

Supplementary Movie 5. Gap Crossing in Isaac-Gym, Small contact surface between gaps (Figure 8-a).

Supplementary Movie 6. Locomotion across small stepping stones (Figure 8-b).

Supplementary Movie 7. Locomotion across a net-like terrain(Figure 8-c).

Supplementary Movie 8. Locomotion across mixed stepping stones (Figure 8-d).
